# Dual beam‐current transformer design for monitoring and reporting of electron ultra‐high dose rate (FLASH) beam parameters

**DOI:** 10.1002/acm2.13891

**Published:** 2023-01-04

**Authors:** Kevin Liu, Allison Palmiero, Nitish Chopra, Brett Velasquez, Ziyi Li, Sam Beddar, Emil Schüler

**Affiliations:** ^1^ Department of Radiation Physics Division of Radiation Oncology The University of Texas MD Anderson Cancer Center Houston Texas USA; ^2^ Graduate School of Biomedical Sciences The University of Texas MD Anderson Cancer Center Houston Texas USA; ^3^ Department of Biostatistics Division of Basic Sciences The University of Texas MD Anderson Cancer Center Houston Texas USA

**Keywords:** beam current transformers, dosimetry, FLASH, toroids

## Abstract

**Purpose:**

To investigate the usefulness and effectiveness of a dual beam‐current transformer (BCTs) design to monitor and record the beam dosimetry output and energy of pulsed electron FLASH (eFLASH) beams in real‐time, and to inform on the usefulness of this design for future eFLASH beam control.

**Methods:**

Two BCTs are integrated into the head of a FLASH Mobetron system, one located after the primary scattering foil and the other downstream of the secondary scattering foil. The response of the BCTs was evaluated individually to monitor beam output as a function of dose, scattering conditions, and ability to capture physical beam parameters such as pulse width (PW), pulse repetition frequency (PRF), and dose per pulse (DPP), and in combination to determine beam energy using the ratio of the lower‐to‐upper BCT signal.

**Results:**

A linear relationship was observed between the absorbed dose measured on Gafchromic film and the BCT signals for both the upper and lower BCT (*R* ^2^ > 0.99). A linear relationship was also observed in the BCT signals as a function of the number of pulses delivered regardless of the PW, DPP, or PRF (*R* ^2^ > 0.99). The lower‐to‐upper BCT ratio was found to correlate strongly with the energy of the eFLASH beam due to differential beam attenuation caused by the secondary scattering foil. The BCTs were also able to provide accurate information about the PW, PRF, energy, and DPP for each individual pulse delivered in real‐time.

**Conclusion:**

The dual BCT system integrated within the FLASH Mobetron was shown to be a reliable monitoring system able to quantify accelerator performance and capture all essential physical beam parameters on a pulse‐by‐pulse basis, and the ratio between the two BCTs was strongly correlated with beam energy. The fast signal readout and processing enables the BCTs to provide real‐time information on beam output and energy and is proposed as a system suitable for accurate beam monitoring and control of eFLASH beams.

## 1 INTRODUCTION

Radiation therapy (RT) is a crucial component of curative cancer therapy, with about 50% of U.S. cancer patients receiving RT as part of treatment.^1^, ^2^ The main purpose of RT is to maximize the therapeutic gain in curing disease while minimizing any associated normal‐tissue complications. One method of accomplishing this is the ultra‐rapid delivery of RT (FLASH RT: mean dose rates ≥40 Gy/s for a total duration of <200 ms^3^, ^4^), which has been shown to spare normal tissues and organs selectively while maintaining a tumoricidal effect in in vivo preclinical models.^4^, ^5^, ^6^, ^7^, ^8^ This phenomenon has been called the “FLASH effect.” FLASH RT represents a fundamentally new paradigm for increasing the therapeutic index of RT relative to the same doses given at conventional (CONV) dose rates (0.01–0.1 Gy/s).^3^, ^9^ The ability to deliver the prescribed dose of radiation to a patient in a shorter overall treatment period with greater normal tissue sparing has enormous implications for the field of RT.^6^, ^10^

One of the major factors limiting the preclinical and clinical use of FLASH RT is the difficulty in obtaining accurate dosimetry and in measuring the irradiation parameters in ultra‐high dose‐rate (UHDR) RT (such as dose, mean and instantaneous dose rate, dose per pulse [DPP], and pulse repetition frequencies [PRFs]) with conventional radiation detectors.^4^, ^11^ Another limiting factor is in the ability to monitor and control the output of the UHDR unit in real time. In a typical linear accelerator, the amount of radiation that is delivered from each pulse is monitored in real time by using dual radiation monitor chambers, that is, a pair of transmission ionization chambers that are installed in the head of the linear accelerator. The function of the transmission chambers is to monitor and control dose, dose rate, beam flatness, beam symmetry, etc., in real time as the beam passes through the chambers. In the case of FLASH beamlines, monitor chambers are not accurate because of the saturation effects caused by the high DPP conditions relevant to UHDR, rendering real‐time dosimetry with clinically relevant detectors an unresolved issue in FLASH RT applications.

Consequently, this study focuses on the use of a beam monitoring device that allows rapid dose monitoring in electron FLASH (eFLASH) beams, which require extensive characterization owing to their rare use in medical physics. The use of beam current transformers (BCTs) for eFLASH beam monitoring is advantageous over transmission ion chambers because they allow real‐time monitoring of the beam output without beam perturbation and saturation effects^12^, ^13^ that are otherwise common in standard beam monitoring approaches with transmission ion chambers.^11^ However, BCTs have two main limitations relative to traditional transmission chambers: (1) their inability to monitor the beam's spatial profile (flatness and symmetry) and (2) their utility in monitoring the beam is limited to charged particles only.^13^

BCTs consist of a conducting wire wrapped around a donut‐shaped object, typically made of iron, and attached to a readout or circuit. The movement of a charged particle through the center of the BCT induces a signal through the electromagnetic principle of induction^14^, ^15^ that is then measured as a pulse signal and correlated to the output of the eFLASH beam. For a pulsed electron linear accelerator in the mega‐electron voltage (MeV) range, the voltage induced in the BCT can be approximated as a function of the electron density of the pulse (greater fluence yields a larger signal) and the cross‐sectional area of the BCT (greater cross sectional area yields a smaller signal).^15^ BCTs are commonly used in high‐energy physics applications but have only recently been suggested for use in UHDR RT applications.^12^, ^16^ Although some investigations^12^, ^16^, ^17^ have been reported on the use of BCTs in CONV and UHDR electron beamlines, to date they have been limited to single BCTs in experimental conditions. Jorge et al.^16^ showed the feasibility of using a BCT for beam monitoring on an experimental eFLASH unit, the Oriatron eRT6 (PMB ALCEN, Peynier France), and Oesterle et al.^12^ described the feasibility of using a BCT for beam monitoring on a FLASH‐capable Mobetron unit (IntraOp, Sunnyvale, CA, USA) by attaching it externally to the head of the Mobetron. Both studies showed the promise of BCTs, demonstrating linearity in signal with dose and the capability of counting and measuring pulses delivered from each accelerator. Based on these results, BCTs were validated and demonstrated their usefulness for providing an independent record and verification of the beam parameters used during the irradiation, and the measured signal in the BCT was found to be correlated with dose to a reference geometry in a manner similar to how monitor chambers are used in conventional clinical linear accelerators.^16^ Thus, BCTs hold great promise for detailed analysis of the physical beam parameters for each pulse delivered via FLASH RT (dose, DPP, pulse width [PW], overall delivery time, mean dose‐rate, instantaneous dose‐rate, and PRF).

The purpose of this work is to introduce a novel use for dual BCTs integrated in the Mobetron unit,^12^, ^16^, ^18^ that is, for real‐time monitoring of beam output and energy as well as measurement of radiation dose in reference geometry in eFLASH beamlines. This study builds on past studies^12^, ^16^ by providing a more comprehensive characterization of the BCTs for different adjustable parameters on the eFLASH Mobetron. The BCTs’ response was characterized for a given number of delivered pulses having different pulse parameters (e.g., pulse number, PW, DPP, and PRF) and in different dosimetric setups and collimation. The ratio of lower‐to‐upper BCT signal was also tested for its usefulness in monitoring beam energy. After this initial characterization here, the usefulness of BCTs can be extended beyond beam and energy monitoring to active control of eFLASH beams in the future.

## 2 MATERIALS AND METHODS

### 2.1 FLASH Mobetron

**FIGURE 1:**
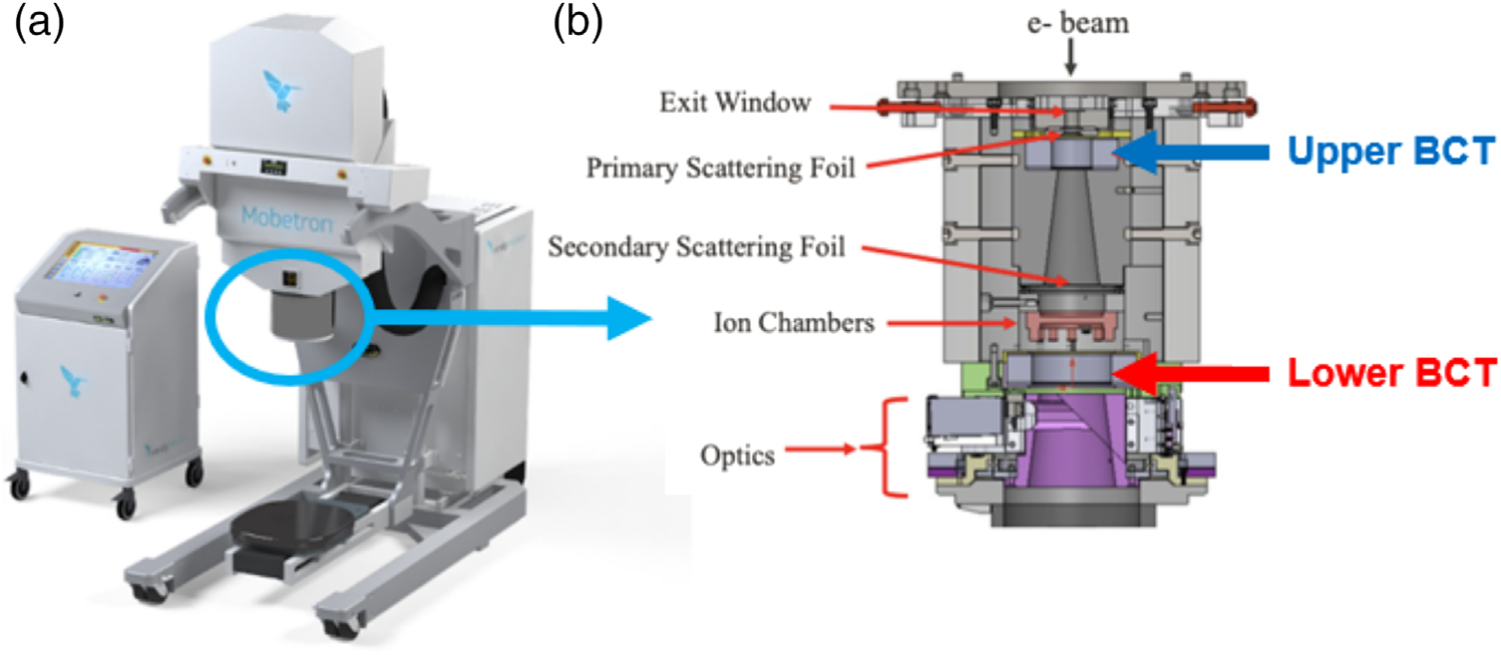
(a) The IntraOp Mobetron system and console. (b) Schematic of the internal components of the head of the Mobetron. The electron pencil beam exits the accelerating waveguide and enters the head of the Mobetron (top). The beam is scattered and traverses two alternating‐current (AC) current transformers (ACCTs) (i.e., toroids or beam current transformers [BCTs]) separated by approximately 13 cm. Before traversing the second BCT, the beam goes through an internal ion chamber, which is used for beam control in CONV mode and for pulse counting in FLASH mode. After the second BCT is the optics equipment that generates the light field, cross hairs, and source‐to‐surface distance (SSD) laser. The beam is collimated by one of two cones of different lengths that are attached to the clamp situated below the optics and by the field size inserts attached to the cone (both not shown).

The FLASH Mobetron (IntraOp, Sunnyvale, CA, USA) (Figure 1a) is a compact commercial linear accelerator that delivers pulsed electron beams at both conventional dose rates (∼10 Gy/min) and UHDRs (>40 Gy/s) for 6‐ and 9‐MeV eFLASH beam energies; it has been commissioned for preclinical purposes and future clinical use as described by Moeckli et al.^17^ In this study, the Mobetron was stored in a shielded vault designed to contain a Varian Clinac 2100 capable of generating photons of energies up to 18 MV. Despite this, a full shielding survey was conducted prior to commissioning of the Mobetron unit.

Our unique Mobetron head design contains built‐in BCTs capable of collecting beam information at different locations in the beam path. The BCTs used in the Mobetron are alternating‐current (AC) current transformers (ACCTs) from Bergoz Instrumentation (Saint‐Genis‐Pouilly, France); they are BCT sensors constructed from a cobalt‐based alloy^19^ connected to their own power supply and external electronic system. Two BCTs are used at different positions within the head of the Mobetron (Figure 1b), one located after the primary scattering foil (upper BCT), and the other after the internal ion chamber and before the optics in the head (lower BCT), thereby allowing redundancy in beam monitoring. The upper BCT is the in‐air ACCT sensor, ACCT‐S‐028, and the lower BCT is the ACCT‐S‐055. The main differences between the two ACCTs are their inner and outer diameters and mass, with the lower BCT having a larger diameter compared to the upper BCT.^19^ The BCTs measure the induced current of the electrons passing through them, thus giving a ‘live’ and non‐destructive temporal readout of the beam.

Before irradiations are delivered with the eFLASH Mobetron, the accelerator performs a 10‐min self‐warmup, and the user then delivers 8000 monitor units (MUs) from the beam in CONV mode as part of the daily protocol. Machine output constancy is then evaluated by delivering a set number of MUs (CONV mode) or a set number of pulses (FLASH mode) to an Advanced Markus ionization chamber (PTW‐Freiburg, GmbH, Freiburg, Germany), which has a reported uncertainty of 2.8% for the saturation model in UHDR modes^20^ at an SSD of 110 cm. This procedure was performed each day to ensure stability of the machine prior to any data collection.

### 2.2 Data processing

**FIGURE 2:**
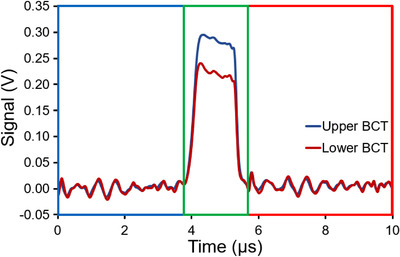
Visualization of acquisition of a pulse signal with Bessel filter and background subtraction applied for a single pulse (typical output trace) from a 9‐MeV eFLASH beam at a pulse width (PW) of 1.2 µs for the upper and lower BCT; the red region corresponds to *M_aft_*, the blue region corresponds to *M_bef_*, and the green region corresponds to *M_raw_* in Equation (2). BCT, beam current transformer. The signal traces for the upper and lower BCTs before and after the pulses are correlated due to interference from the power supply.

Users delivering eFLASH beams with the Mobetron can specify certain parameters such as the width of the pulse to be delivered (PW, in µs) and the repetition frequency for each pulse (PRF, in Hz). Here, the signal from each BCT after a pulsed delivery was acquired by using a PicoScope 5000 Series FlexRes Oscilloscope (Pico Technology, St Neots, Cambridgeshire, UK) operating with 14‐bit resolution and sampling rate of 125 MS/s (Mega Samples per second) in rapid acquisition mode based on vendor recommendations. Data collected from the BCTs underwent multi‐tier processing, as described below, to assist with characterization of the signals. The signals were forward‐backward filtered by passing a Bessel function^21^, ^22^ over the waveform via convolution as shown in Figures S1 and 2. A custom pulse‐finding algorithm was written to take advantage of the relatively high baseline noise of BCT signals by looking for the locations where the signal is equal to a threshold value (e.g., 100 mV), and then locating the first local minima before (t_1_) and after (t_2_) the two locations of the threshold value as shown in Figure 2. The pulse is defined as the signal between the two locations, t_1_ and t_2_. The integral of the BCT signal related to the individual pulses is modified by subtracting the baseline noise before and after the pulse delivery received from each BCT to consider changes in noise (Figure 2).

(1) $${\;M}_{\mathrm{raw}}=\int_{t_{1}}^{t_{2}}S^{\prime }\left(t\right)\mathrm{dt}\;\left[\mu V\cdot s\right]$$

This process is described in Equations (1) and (2), where $M_{raw}$ is the integrated signal $S^{\prime }\left(t\right)$ obtained from the BCT, $M_{bef}$ is the integrated noise before the pulse, and $M_{aft}$is the integrated noise after the pulse. The bounds on $M_{raw}$ are set by using an in‐house‐written peak‐finding algorithm. The bounds on $M_{bef}$ and $M_{aft}$ are chosen based on the results of the pulse‐finding algorithm and scaled to the window of the $M_{raw}$ signal.

(2) $$M\;=M_{\mathrm{raw}}\;-\frac{1}{2}\left(M_{\mathrm{bef}}+M_{\mathrm{aft}}\right)\left[\mu V\cdot s\right]$$

### 2.3 Dose linearity measurements

Dosimetric evaluations were performed using GafChromic EBT3 film (Ashland Specialty Ingredients, Bridgewater, NJ, USA) to measure dose in a fixed geometry under the following reference conditions: a source‐to‐surface distance (SSD) of 42 cm, using an applicator with a 10‐cm diameter insert and a 5‐cm air gap between the insert and the surface (thereafter referred to as our standard dosimetric setup). Film was selected as the dosimeter for dose measurements owing to its dose‐rate independence up to 9 × 10^12^ Gy/s.^4^, ^23^ The dose delivered to a single location was scored at the depth of maximum dose (d_max_) in solid water for a 9‐MeV eFLASH beam. The PW and PRF were kept constant at 1.2 µs and 120 Hz, with a DPP of 0.75 Gy. To evaluate the linearity of dose delivered from the eFLASH beam with the measured BCT signal, we delivered 1, 5, 10, 20, 30, 40, and 50 pulses to the EBT3 films placed at d_max_. This experiment was repeated three times at all of the above pulse numbers. A summary of the fixed and variable experimental parameters performed are described and tabulated in Table 1.

**TABLE 1:** Summary of the fixed and variable experimental parameters within each characterization study

Type of study	Fixed parameters	Variable parameters
Dose linearity	Source‐to‐surface distance Pulse width Pulse repetition frequency Beam energy	Number of pulses
Pulse width dependence	Source‐to‐surface distance Pulse repetition frequency Beam energy	Number of pulses Pulse width
Pulse‐by‐pulse stability	Source‐to‐surface distance Number of pulses	Pulse width Pulse repetition frequency Beam energy
Pulse repetition frequency	Source‐to‐surface distance Pulse width Beam energy	Number of pulses Pulse repetition frequency
Energy monitoring	Source‐to‐surface distance Number of pulses Pulse repetition frequency Pulse width	Beam energy
Electron scatter	Number of pulses Pulse repetition frequency Pulse width Beam energy	Source‐to‐surface distance Types of scattering surfaces

At 24 h after irradiation, EBT3 films, stored in the dark at a 22°C ambient temperature environment, were scanned with an Epson 10000XL flatbed scanner in transmission mode, landscape orientation, 48‐bit color, 72 dpi, and without color correction. Film placement on the scanner was made consistent with the aid of a cardboard cutout. The scanned film data were analyzed by measuring the net optical density (netOD) of the irradiated films relative to an unirradiated (0 Gy) film from the same batch. The red channel component of the scanned image was analyzed by using ImageJ and MATLAB in acquiring the mean pixel value for each irradiated film used. The mean pixel value measured for the three films in each dose group were averaged to acquire an averaged mean pixel reading. The netOD reading was acquired by taking the base‐10 logarithmic ratio of the averaged mean pixel reading from the unirradiated films, $Pixel_{background}$, with the averaged mean pixel reading from the films irradiated to an absorbed dose, $Pixel_{dose}$:

(3) $$\mathrm{netOD}\;=\;\log10\left(\frac{Pixel_{background}}{Pixel_{dose}}\right)$$

The change in netOD of the film was correlated to dose by using a dose calibration curve from the same batch of film irradiated to known doses ranging from 0.5 to 50 Gy,^24^ as performed in previous studies.^25^, ^26^ Moreover, each experiment that involved film and the signal measured on BCTs was done in triplicate and reported as the averaged readings with error bars depicting one standard deviation from the triplicate measurements. In some figures, the error bars are hidden behind the measurement point because of their relatively small value.

### 2.4 Pulse width (PW) dependence

To determine whether the BCT response was influenced by PW, the signal measured from the BCTs were collected during deliveries of 1, 2, 5, 10, 20, 50, 100, and 200 pulses of a 9‐MeV eFLASH beam at PWs of 1, 1.2, 2, 3, and 3.6 µs, with a constant PRF of 120 Hz (Table 1). The pulse delivery for linearity measurements was done by first selecting the modifiable beam setting (e.g., PW) and then delivering 1–200 pulses in triplicate for each pulse number before selecting the next beam setting. This delivery sequence was performed in subsequent sections of this study as well. The PWs were set by the manufacturer as the distance between the 50% reference level (the full width at half maximum FWHM) as defined by IEEE standards,^27^ and was confirmed by us. Two‐hundred pulses were the highest pulse number selected due to the manufacturer setting 200‐pulses as the upper limit for the number of pulses that can be delivered in a single delivery. The BCT signals from three measurements were averaged to produce the mean signal from a given number of pulse deliveries. Beams were delivered without collimation, with no object obstructing the beam aside from the beam stopper in Figure 1a. This same setup was used in subsequent investigations of the BCT response as a function of pulse number and PRF.

The relationship between BCT response and DPP was investigated by modifying all the aforementioned PWs at a constant PRF of 120 Hz and comparing the response to what was measured on EBT3 film. The irradiation set‐up involved percent depth dose (PDD) measurements with the eFLASH beams by placing EBT3 film (4.5 × 9.0 cm^2^) in a water tank with a film holder at a 2‐degree tilt, using the dosimetric setup described by Arjomandy et al.,^28^ with an SSD of 42 cm, and a 5‐cm air gap between the exit window and water surface. A total of 30 pulses were delivered to films at PWs of 1 and 1.2 µs; 20 pulses were delivered at a PW of 2 µs; and 15 pulses were delivered at PWs of 3 and 3.6 µs. Three films were irradiated at each PW investigated. After irradiation, the EBT3 film was scanned at 150 dpi to obtain the netOD, at a high enough spatial resolution, which was converted to a depth‐dose curve using the dose conversion method described above. The dose at d_max_ was obtained from each scanned film corresponding to their PW and were normalized to the number of pulses delivered. The DPP values were plotted against the BCT response per pulse.

### 2.5 Pulse‐by‐pulse stability

To evaluate the performance of the eFLASH Mobetron accelerator with the number of pulses delivered, 200 pulses were delivered with the following pulse settings: 1.2 µs/30 Hz, 1.2 µs/120 Hz, 2 µs/120 Hz, and 3 µs/120 Hz (Table 1). The 6 MeV eFLASH beam was also used to examine a single pulse setting of 1.2 µs/120 Hz for comparison with the pulse structure of the 9‐MeV eFLASH beam. At any given pulse setting, three 200‐pulse deliveries were performed and the BCT response was analyzed individually for each delivered pulse. To examine variation in the measured pulse signal at a specific pulse number between different deliveries (inter‐delivery pulse variation), or the variation of the measured pulse signal over the entire sequence of pulses from a single beam delivery (intra‐delivery pulse variation), the coefficient of variation (COV) metric was used. The COV was defined in one of two ways: as the percent value of the standard deviation divided by the mean value of the pulse signal measured at the nth sequence in the 200‐pulse train for three beam deliveries (inter‐delivery pulse COV); or as the standard deviation divided by the mean value of the signal measured from a single 200‐pulse delivery (intra‐delivery pulse COV). The inter‐delivery pulse COV quantifies the extent of variation between pulses at a specific position in the sequence of the 200 pulses in multiple deliveries; the intra‐delivery pulse COV quantifies variation between pulses from the same delivery.

### 2.6 Pulse repetition frequency (PRF) dependence

To determine whether the BCT response is influenced by PRF, the signal measured from the BCTs were collected after delivery of 1, 2, 5, 10, 20, 50, 100, and 200 pulses from the 9‐MeV eFLASH beam at PRFs of 30, 60, 90, and 120 Hz and a constant PW of 1.2 µs (Table 1). All measurements were performed in triplicate and averaged to produce the mean signal at each delivered pulse number from a given number of pulse deliveries.

The potential influence of PRF on radiation dose was investigated by modifying all the aforementioned PRFs at a constant PW of 1.2 µs. The irradiation set‐up involved the same PDD described previously. A total of 30 pulses were delivered to film at a PW of 1.2 µs and at PRFs of 30, 60, 90, and 120 Hz. Three films were irradiated at each PRF investigated. After irradiation, the EBT3 film was scanned at 150 dpi to obtain the netOD, at a high enough spatial resolution, which was converted to a depth‐dose curve using the dose conversion method described above. The dose at d_max_ was obtained from each scanned film corresponding to its PRF. The measured BCT signal, averaged from three deliveries, was normalized to the dose measured at d_max_ at each PRF setting. The PRF settings were plotted against the BCT signal per unit dose value.

### 2.7 Energy monitoring

To investigate the response of the BCT as a function of the energy, the energy of the beam was modified by manipulating the electron gun grid and the RF shorts position in the waveguide (i.e., energy switch) while keeping other settings constant; the pulse flatness was recovered by tuning the automatic frequency control voltage (Table 1). Modifying the grid voltage affects the number of electrons in the beam and modifying the RF shorts changes the resonance of the beam and thereby affects the average energy per electron. In tuning these parameters, we affected the acceleration potential in the waveguide downstream, causing the electron bunch to move slightly off‐sync from the RF wave and thereby causing the energy of the beam to vary with each modification. For each incremental modification, 40 pulses were delivered onto film placed in our film water tank, while BCT data were acquired simultaneously. The depth of 80% of maximum dose (R_80_) and depth of 50% of maximum dose (R_50_) were obtained from each scanned film, corresponding to their incremental modification of the grid level. These values were then plotted against the lower‐to‐upper BCT ratio values. Moreover, the upper BCT signal from each irradiation was normalized to the dose delivered at d_max_ from the PDD measured on film and was plotted as a function of the measured R_50_.

To understand the statistical power of detecting a shift in R_50_ using these BCT observations, we first fit linear and quadratic models using R_50_ as the covariate and the BCT ratio as the outcome. The absolute mean prediction error for the quadratic model (0.016) was smaller than the linear model (0.021), and thus we used the quadratic model as the final predictive model. Because the observation error of BCT ratio depends on the value of R_50_, we designed a simulation study to compute the actual statistical power. In the simulation study, we first estimated the association of the BCT ratio with the R_50_ level by using a quadratic linear regression model. We then resampled the BCT ratio and incorporated normally distributed measurement noises to the observation with the mean and variance estimated from the k‐nearest neighboring observed noises, with *k* = 3 to mimic the noise in each location. This was performed by generating 10 repeated measurements in adding the prediction from the quadratic linear regression model with the simulated observation noise for each R_50_ of interest. Two‐sample t‐tests were performed at each R_50_ level to compute the p‐values between the values of R_50_ and the BCT ratio. Any *p*‐values smaller than 0.05 were deemed to be statistically significant and the scenario was counted towards the power calculation. To provide robust conclusions, the results were averaged from 1000 Monte Carlo datasets for final statistical power estimations.

### 2.8 Electron scatter dependence

To examine how the effects of electron scatter may influence the BCT signal and to determine the need for output correction factors, BCT measurements were collected for different dosimetric setups and configurations for the 9‐MeV eFLASH beam (Table 1). Seven different setups were used to examine this effect: (1) an uncollimated open‐beam configuration in which no object was present in the beamline other than the beam‐stopper; (2) an open‐beam configuration with a 10‐cm collimator attached to an applicator; (3) an open‐beam configuration with a 2.5‐cm collimator attached to the same applicator; (4) the standard dosimetric configuration (as described in Section 2.2), with a 5‐cm air gap between the end of a 10‐cm collimator and solid water; (5) the standard dosimetric configuration with a 5‐cm air gap between the end of a 2.5‐cm collimator and solid water; (6) an uncollimated beam with solid water placed directly underneath the exit window of the Mobetron head; and (7) an uncollimated beam with lead placed directly underneath the exit window. Five 10‐pulse irradiations were conducted in each setup, and the signals from the BCTs were collected and compared.

## 3 RESULTS

### 3.1 Dose linearity

**FIGURE 3:**
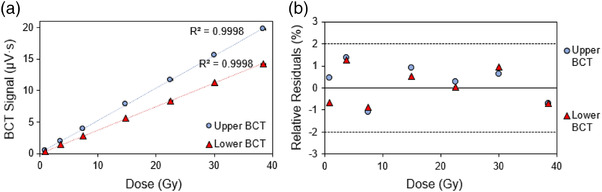
(a) The measured signal from the upper and lower beam current transformers (BCTs) as a function of dose at a dose per pulse (DPP) of 0.75 Gy with (b) showing the relative residuals (the dotted lines represent ± 2% relative residuals and the solid line is the 0% boundary). Error bars represent one standard deviation from three irradiations

The measured signals from the upper and lower BCTs as a function of absorbed dose are shown in Figure 3. A linear relationship between the absorbed dose and the measured BCT signal was observed for both BCTs (Figure 3a) with the relative residuals (%RR) being less than 2% (Figure 3b). Both BCTs had high linearity with increasing delivered dose having an *R* ^2^ of >0.999.

### 3.2 Pulse width (PW) dependence

**FIGURE 4:**
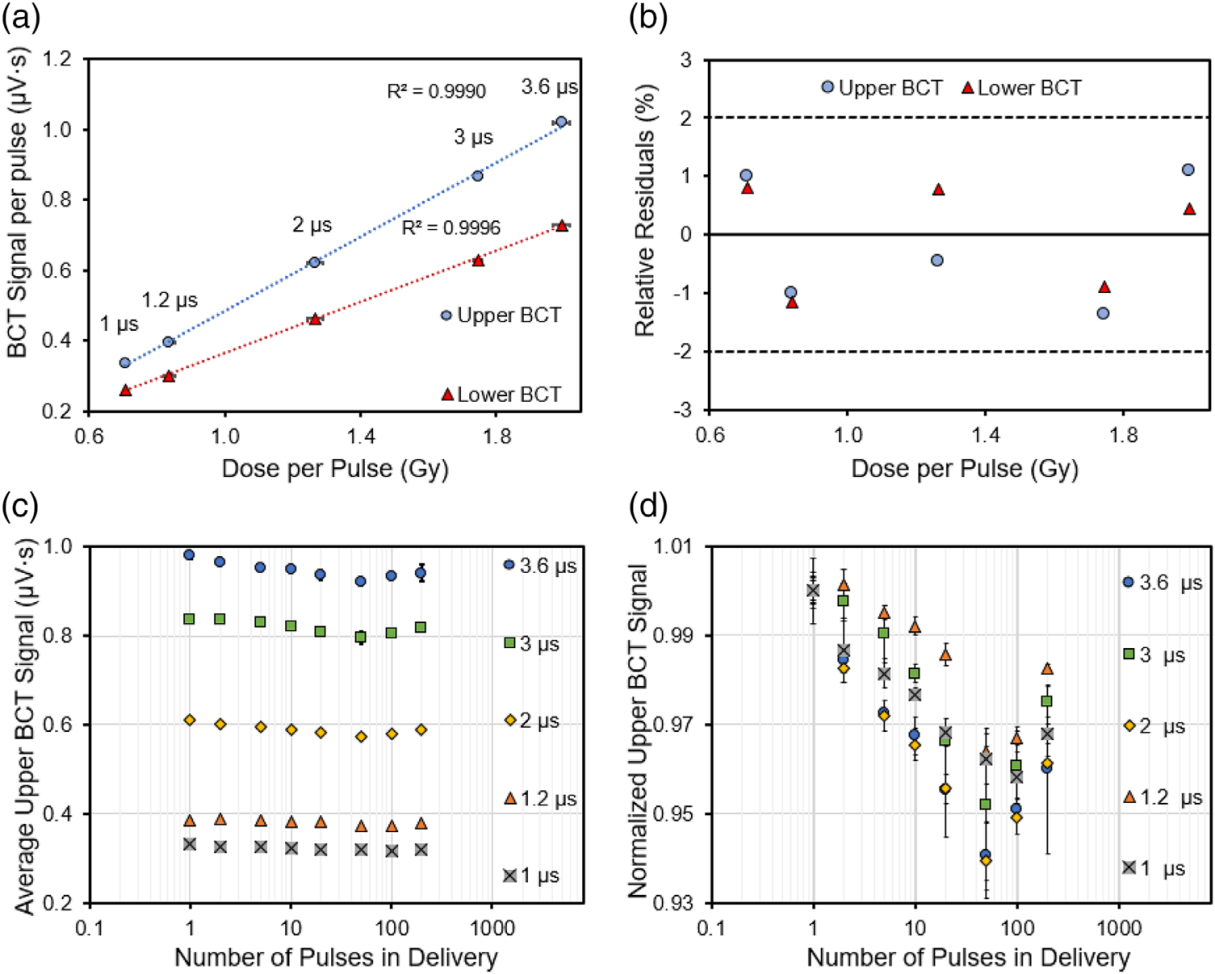
(a) The upper and lower BCT signal per pulse as a function of dose per pulse (DPP) (0.7–2 Gy/pulse) using PW settings of 1–3.6 µs. (b) The relative residuals for the measured upper and lower BCT signal as a function of DPP with the dotted lines representing ± 2% relative residual and a solid line at the 0% boundary; (c) The measured upper BCT signal averaged from three measurements and normalized to the number of pulses delivered; and (d) the upper BCT signal normalized to the signal measured at the one‐pulse delivery. The error bars represent one standard deviation from three measurements at a PRF of 120 Hz, with the 9‐MeV eFLASH beam

Figure 4a illustrates the linear relationship between the measured DPP with the BCT signal per pulse for both the upper and lower BCT, and Figure 4b shows that the relative residuals are <2% for the BCT signal plotted as a function of the DPP. Figure 4c shows the upper BCT signal from a given number of pulse deliveries normalized to the number of pulses delivered with the 9‐MeV eFLASH beam. This panel depicts PWs ranging from 1 to 3.6 µs at a standard PRF of 120 Hz. Figure 4d and Table S1 show the averaged upper BCT signal per pulse values from Figure 4c normalized to the averaged upper BCT signal per pulse from delivery of a single pulse to illustrate the relative variation in BCT signal per number of pulses delivered at each examined PW.

The BCT signal per pulse measured for a given number of pulses declines with each subsequent pulse number until about 50 pulses, after which the measured BCT signal per pulse increases with each subsequent pulse number delivered (Figure 4c,d). The relative difference between the measured BCT signal per pulse between the 1st pulse and the 50th pulse in Figure 4d was as high as 6% depending on the PW, with 2 and 3.6 µs having the highest relative difference at the 50th pulse, at a PRF of 120 Hz. Despite this difference, when the measured BCT signal was plotted as a function of the number of pulses delivered and the measured BCT signal per pulse was assessed as a function of the DPP for each PW, the linearity remained, with an *R* ^2^ of ≥ 0.999 (Figures 4 and S2).

### 3.3 Pulse‐by‐pulse stability

**FIGURE 5:**
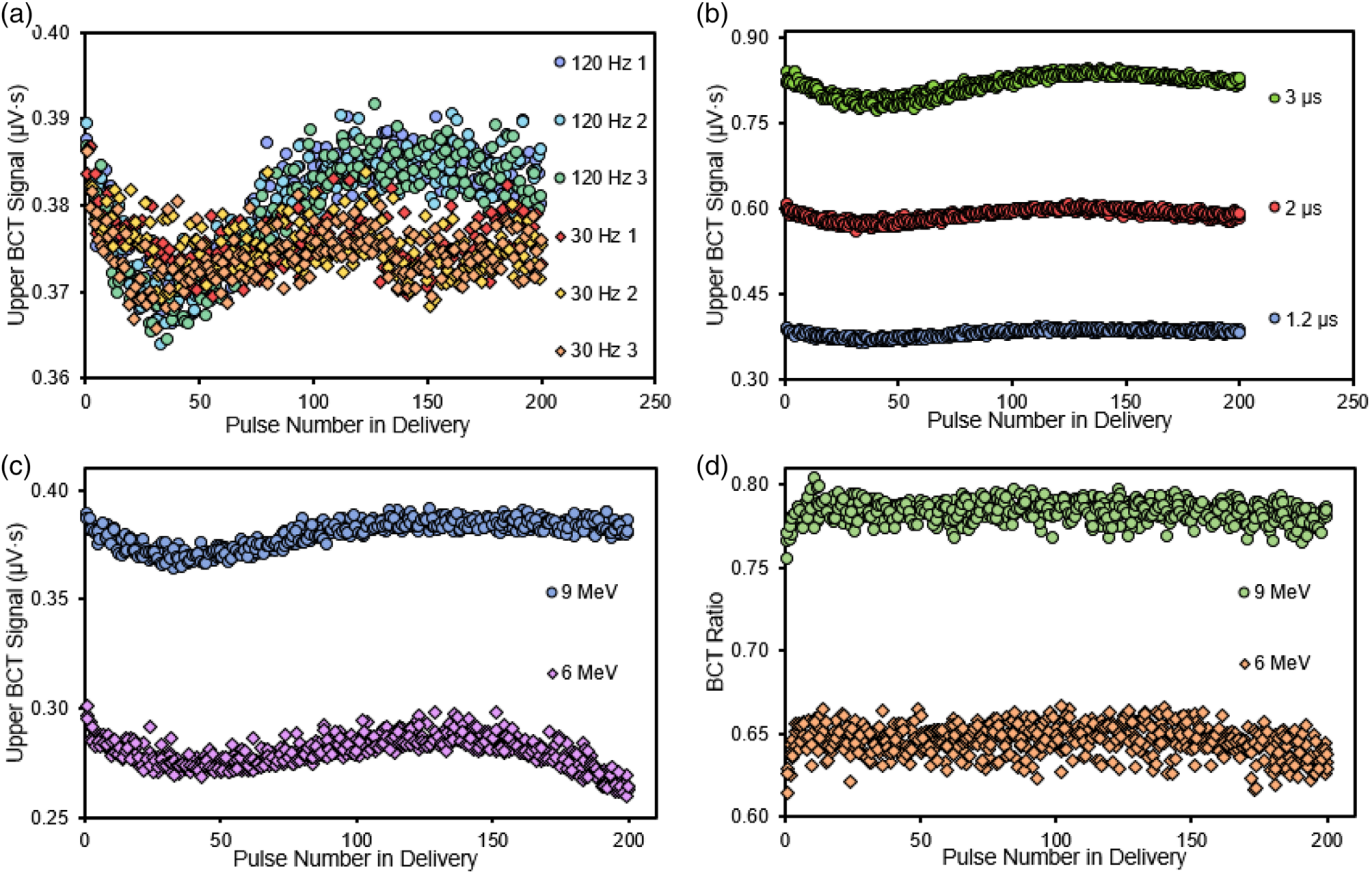
(a) Upper BCT signal for each individual pulse delivered during three 200‐pulse deliveries at a PW of 1.2 µs and PRFs of 120 Hz (circles) and 30 Hz (diamonds) with the 9‐MeV eFLASH beam; each of the three deliveries is denoted by a different color. (b) Upper BCT signal for the 9‐MeV eFLASH beam at a PRF of 120 Hz and PWs of 1.2, 2, and 3 µs; data is combined from three deliveries. (c) Upper BCT signal at a PW of 1.2 µs and a PRF of 120 Hz for 6‐MeV and 9‐MeV eFLASH beams; data is combined from three deliveries. (d) Ratio of lower‐to‐upper BCT signal for each pulse delivered at a PW of 1.2 µs and a PRF of 120 Hz from 6‐MeV and 9 MeV eFLASH beams; data is combined from three deliveries

In this section, the utility of BCTs to monitor the pulse‐by‐pulse stability and performance of the accelerator was evaluated as a function of PRF, PW, and energy. Figure 5 shows the individual pulses delivered from the accelerator in three separate 200‐pulse deliveries at different PW, PRF, and pulse energy configurations.

Figure 5a shows the measured signal in individual pulses generated at a PW of 1.2 µs and at PRFs of 30 and 120 Hz for three 200‐pulse deliveries. For both PRFs investigated, the measured pulse signal in the upper BCT decreased for each subsequent pulse delivered up until the 40th pulse. Beyond the 40th pulse, at 120 Hz, the measured signal for each delivered pulse increased and recovered to a certain value (∼0.385 µV·s), whereas at 30 Hz, the measured signal for each delivered pulse stabilized at about 0.375 µV·s (an offset of approximately 3% between the measured individual pulse signals for 30 and 120 Hz at the 200th pulse delivery).

Figure 5b depicts the measured signal in individual pulses generated in three 200‐pulse deliveries for PWs of 1.2, 2, and 3 µs at a standard PRF of 120 Hz. The spread in the measured pulse signal at a given pulse number for the three measurements delivered at each PW was observed to be higher for the larger PWs, with a maximum inter‐delivery pulse COV of 1.55% for 1.2 µs, 1.73% for 2 µs, and 1.89% for 3 µs; corresponding maximum intra‐delivery pulse COVs were 1.72% (1.2 µs), 1.95% (2 µs), and 2.57% (3 µs).

Figure 5c juxtaposes the measured signal in individual pulses generated for a 6‐MeV and a 9‐MeV eFLASH beam at a PW of 1.2 µs and a PRF of 120 Hz for three 200‐pulse deliveries. At the same PW and PRF, variation in the measured pulse signal was greater at a given number of pulses for the 6‐MeV eFLASH beam than for the 9‐MeV eFLASH beam in the three beam deliveries, with the maximum inter‐delivery pulse COV being 3.28% for the 6‐MeV beam and 1.55% for the 9‐MeV beam, with corresponding maximum intra‐delivery pulse COVs of 2.52% and 1.72%.

In Figure 5d, the ratio of lower BCT‐to‐upper‐BCT for the 6‐MeV and 9‐MeV eFLASH beams, for three 200‐pulse deliveries at a PW of 1.2 µs and a PRF of 120 Hz, is shown. Notably, the BCT signal for the first pulse delivered by both the 6‐MeV and 9‐MeV eFLASH beams was 2% to 4% lower than the mean BCT ratio in the 200‐pulse train. This was caused by the variation in measured output from the first pulse relative to subsequent pulses as observed in Figure 5a–c. However, the lower‐to‐upper BCT ratio remained fairly constant throughout the entire pulse train, with the intra‐delivery pulse COV in the lower‐to‐upper BCT ratio for 200 pulses in a single delivery being <1% (0.72%–0.85%) for all three beam deliveries in the 9‐MeV eFLASH beam and <1.5% (1.41%–1.46%) in the 6‐MeV eFLASH beam. The average BCT ratio in three 200‐pulse deliveries was 0.783 in a 9‐MeV eFLASH beam and 0.646 in a 6‐MeV eFLASH beam. The inter‐delivery pulse COV for the average BCT ratio between the three 200‐pulse deliveries was 0.23% and 0.19% for the 6‐ and 9‐MeV eFLASH beam respectively.

### 3.4 Pulse repetition frequency (PRF) dependence

**FIGURE 6:**
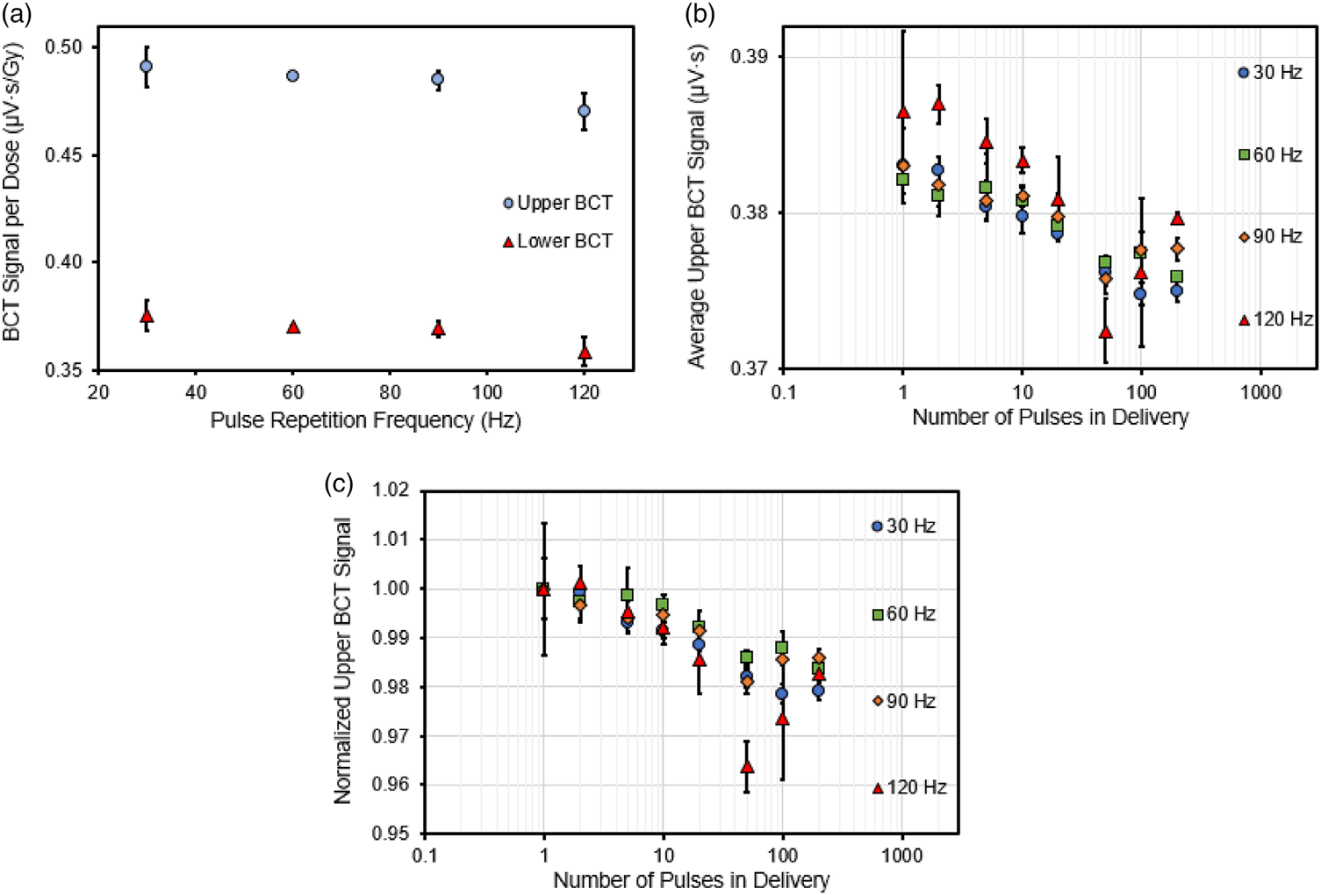
(a) The upper and lower BCT signal per unit dose as a function of PRF using a dose per pulse (DPP) of 0.80 Gy. (b) The measured upper BCT signal for different pulse repetition frequencies (PRFs) normalized to the number of pulses delivered. (c) The upper BCT signal normalized to the signal measured at the first pulse. The error bars represent one standard deviation from three measurements. Data acquired with the 9‐MeV eFLASH beam with a PW of 1.2 µs

Figure 6a plots the mean BCT signal, from three deliveries, normalized to dose at different PRFs. For both the upper and lower BCTs, it was found that the BCT signal per dose decreases at higher PRFs with a maximum difference for both the upper and lower BCT being 4.4% and 4.8% respective between the 30 and 120 Hz PRF setting. Figure 6b depicts the average signal from three BCT readings for 1–200 delivered pulses, normalized to their respective delivered number of pulses. In Figure 6c, values from Figure 6b are normalized for each PRF to the average BCT reading delivered from deliveries with only one pulse. Table S2 also shows the values in Figure 6b, normalized to the average BCT signal per number of pulses delivered from three readings in the delivery of a single pulse for the listed PRFs.

The BCT signal per pulse, measured for a given number of pulses, was found to decline with each subsequent pulse number until about 50 pulses. When the PRF was 30–90 Hz and the PW was held at 1.2 µs, the BCT signal per pulse beyond 50 pulses tended to stabilize at about 0.375 µV·s to 0.380 µV·s, a finding that differs from the pulse structure observed at a PRF of 120 Hz. The relative difference between the measured BCT signal per pulse for different PRFs at a PW of 1.2 µs was as high as 2%–4%. It is important to note that the relative difference between the measured BCT signal per pulse is as high as 2% for the PRF setting of 30, 60, and 90 Hz while the 120 Hz setting demonstrated the largest relative difference of 4% at the 50th pulse delivery. Despite this difference, the measured BCT signal plotted as a function of the number of pulses delivered for each PRF was still linear, with an *R* ^2^ of >0.999 (Figure S3).

### 3.5 Energy monitoring

**FIGURE 7:**
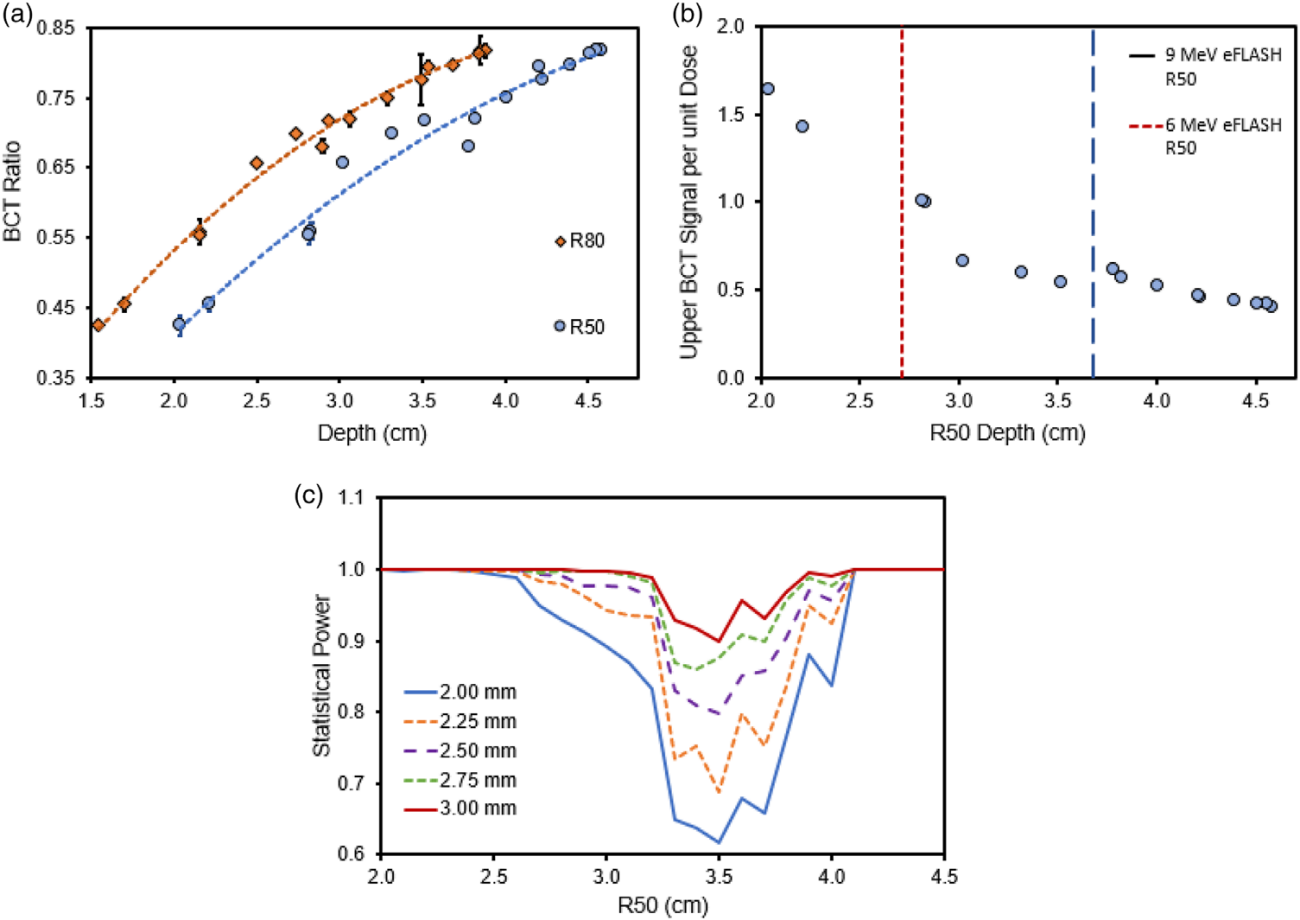
(a) The ratio of the lower‐to‐upper BCT as a function of the R_50_ and R_80_ depths. (b) The upper BCT signal normalized to the measured dose at d_max_ as a function of the beam energy (represented by R_50_). The vertical red and blue lines in (b) are the R_50_ values of our standard 6‐ and 9‐MeV eFLASH beams, respectively. Error bars represent one standard deviation from the delivered pulses measured on the BCTs and film. (c) The statistical power based on the Monte Carlo datasets in detecting shifts of 2, 2.25, 2.5, 2.75, and 3‐mm in the measured R_50_ using the detected shifts in the lower‐to‐upper BCT ratio

Figure 7a illustrates the lower‐to‐upper BCT ratio measured at different beam energies with a quadratic fit; these energy modifications were quantified by using the electron beam parameters R_50_ and R_80_. Figure 7b plots the BCT signal normalized to dose as a function of the eFLASH beam energy (R_50_) with the 6 and 9 MeV eFLASH R_50_ values for reference. The BCT signal per dose is shown to decrease with increasing energy. Figure 7c summarizes the statistical power using the BCT ratio to detect the distance shift of R_50_, with possible distance shifts ranging from 2‐mm to 3‐mm. The detection ability of BCT ratio depends on the R_50_ values of interest. The middle range of the R_50_ values in particular tend to be associated with higher observation errors and thus have less power to detect a fixed distance shift in R_50_. Next, the results show that statistical power was sufficient (>90%) to detect an R_50_ distance shift of 2‐mm at the lower and upper ends of the range of interest, that is, R_50_ < 3 cm and R_50_ > 4 cm. For the middle range of R_50_ values, the statistical power was >80% for detecting a distance shift of 2.5 mm or more.

### 3.6 Electron scatter dependence

**FIGURE 8:**
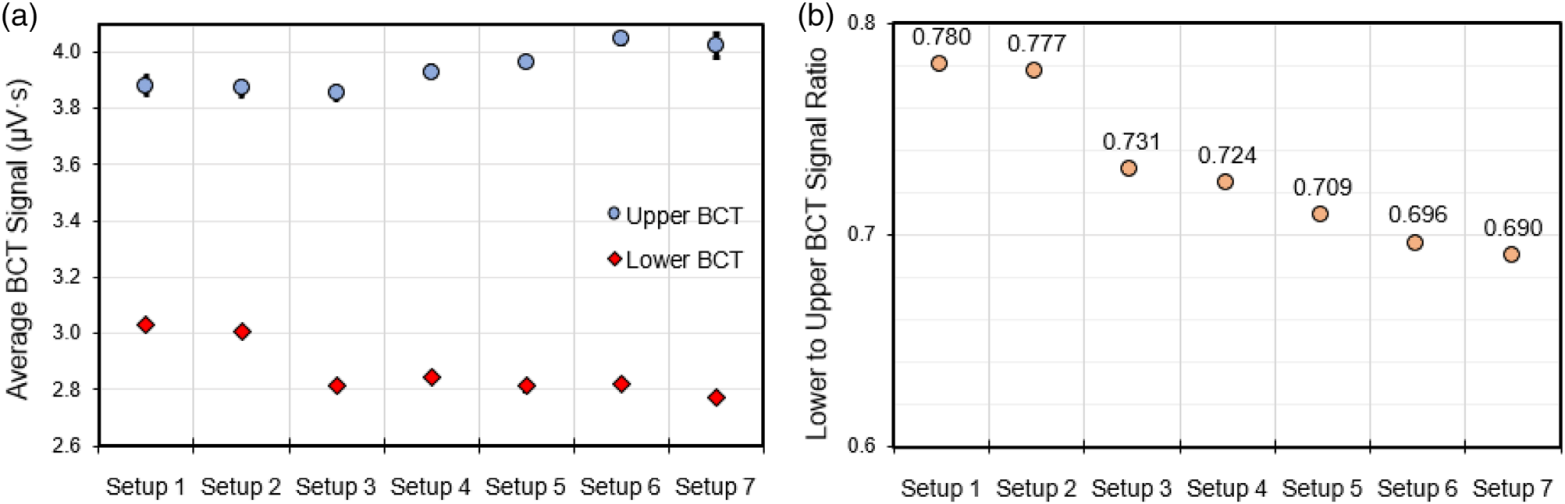
(a) The measured upper and lower BCT signal from a delivery of 10 pulses averaged over five deliveries under the indicated experimental conditions affecting electron back scatter: Setup 1: Open‐beam configuration uncollimated; Setup 2: Open‐beam configuration with 10 cm collimation; Setup 3: Open‐beam configuration with 2.5 cm collimation; Setup 4: Standard dosimetric configuration with 5‐cm air gap between the end of 10 cm collimator and solid water; Setup 5: Standard dosimetric configuration with 5‐cm air gap between the end of 2.5 cm collimator and solid water; Setup 6: Uncollimated beam with solid water directly underneath exit window; and Setup 7: Uncollimated beam with lead directly underneath exit window. (b) The ratio of the lower‐to‐upper BCT signal in the indicated conditions. Error bars represent one standard deviation from the average signal measured on the BCT from five 10‐pulse deliveries

Figure 8a shows the upper and lower BCT signal measured under different scatter conditions and collimator configurations. The ratio of the lower‐to‐upper BCT signal values for each of these conditions is shown in Figure 8b. Adding collimation and modifying the dosimetric setup were found to influence both the upper and lower BCT signal, given that the same number of pulses were delivered. In general, as the beam became more collimated and as the scattering surface is moved closer to the exit window, the upper BCT signal varies by 1% to 2%, while the lower BCT signal decreases by up to 12% relative to the open beam uncollimated dosimetric setup.

## 4 DISCUSSION

Previous studies have described the response of a single BCT placed at the exit/exterior of the accelerator as a function of the measured charge delivered to an Advanced Markus chamber; these reports demonstrated linearity in the BCT signal with the measured charge‐dose from the ion chamber.^12^, ^16^ Oesterle et al.^12^ placed a BCT sensor at the exit of a Mobetron capable of UHDR irradiation, and Jorge et al.^16^ placed a BCT sensor at the exit of an Oriatron to measure the beam current at the exit window for CONV and FLASH electron beams. CONV irradiation, delivered with a 30‐Hz repetition frequency and greatly reduced beam current in the eFLASH Mobetron used in this study, was not evaluated because the BCTs that were used cannot measure the low dose‐per‐pulse irradiation used in CONV mode. Moreover, BCTs have already been evaluated for CONV irradiation elsewhere^12^, ^16^ and the incorporation of internal ion chambers in the eFLASH Mobetron by the manufacturer allows control and monitoring of the electron beam at conventional dose rates. The BCTs used in the aforementioned studies revealed that overlapping signals between the eFLASH and conventional beams for the measured PWs and PRFs were both nearly identical to the machine parameters used for each measurement, demonstrating the utility of BCTs in monitoring the accelerator performance and output. The BCT signals were also linear with accumulated dose measured in the ion chamber across the investigated PWs (0.5–4.0 µs,^12^ 1.0–4.0 µs^16^); PRFs (5–90 Hz,^12^ 5–250 Hz^16^); and energies (6‐ and 9‐MeV,^12^ and 5.4‐MeV^16^). These results are foundational in our investigation of BCTs to monitor the output of eFLASH beams in real time.

The current study builds on this previous work by investigating the utility of dual‐BCTs incorporated into the interior of the eFLASH accelerator, designed for preclinical and clinical use, for redundant monitoring of a variety of modifiable beam parameters and beam energy. Because the eFLASH Mobetron was designed for a clinical context, it is important to evaluate the number of pulses delivered, the beam energies, PW, PRF, and DPP for a variety of potential irradiation sites and prescription doses in the context of beam monitoring with BCTs. The ideal settings for a given clinical setup have yet to be determined, because this research is ongoing; for that reason we evaluated all the relevant beam settings available and provided a comprehensive investigation of the utility of BCTs as a beam monitoring solution for eFLASH beams. The BCT signals were found to be linear with respect to the dose and DPP delivered, and the two BCTs were demonstrated to be capable of functioning as beam monitors independent of one another to monitor the dose delivery of an eFLASH beam after each pulsed delivery, thereby taking on the role of the traditional transmission chambers embedded in standard clinical linear accelerators.

Moreover, the BCTs demonstrated their utility in evaluating the performance of the eFLASH accelerator on a pulse‐by‐pulse basis. This was validated in Figures 4, 5, 6, where individual signals from each pulse delivered were measured and plotted for different PRF, PW, and energy settings, with some of these settings corelated to dose measured on film. These figures illustrate that different PRF settings and different beam energies affect the pattern of individual pulses delivered in a pulse train by the eFLASH beam; that electron backscatter affects the signal measured in the lower and upper BCT (the BCT ratio); and that larger PWs yield a greater spread in the measured BCT signal for multiple deliveries (inter‐delivery pulse variation). This capability for monitoring the pulse‐by‐pulse behavior of the accelerator would allow the user to define the absorbed dose under established dosimetric conditions based on the output measured by the BCT, and to track the dose delivered based on the measured BCT signal and the dose conversion factor specific to the dosimetric setup.

Given that we have established the linearity of the cumulative BCT signal for a given number of delivered pulses with absorbed dose, we were also able to demonstrate linearity in the BCT signal from a given number of pulses for different PW and PRF parameters. These results provide a more thorough picture of the pulse structure of an eFLASH beam by using BCTs as opposed to any other commercially available detector. From this we can see that BCTs are a useful tool for evaluating the performance of the eFLASH accelerator in terms of pulse‐by‐pulse stability, PW, PRF, and scatter. Small deviations in the individual and in the cumulative pulse signal at different beam parameters were also thoroughly visualized with the BCT, allowing the user to observe any instabilities attributed to the accelerator performance and account for these instabilities. Our findings further show that the signal from each individual pulse can be correlated to dose given the proper calibration, demonstrating the utility of BCTs for providing real‐time dose monitoring of an eFLASH beam without the effects of beam perturbation or detector saturation. We further found that the BCTs have an uncertainty on par with EBT3 film, which does limit the current study, as the ideal case would be to test a new instrument against an instrument with greater accuracy and reproducibility. The uncertainty of film for dose measurements in UHDR beams has been reported as being 2%^12^, ^16^ related to FLASH dosimetry, whereas the overall uncertainty of EBT3 film in UHDR beams has been characterized as <4% (*k* = 2),^29^ with the quality of dose‐rate independence.^23^, ^29^ Other methods to evaluate the performance of BCTs were not evaluated in this study, such as comparing the BCT signal against the integrated charge measured on a capacitor.^30^, ^31^, ^32^ Because dosimetry in FLASH is still in its early stages, Gafchromic EBT3 film remains the standard dosimeter against which other detectors are calibrated in many FLASH dosimetry studies.^4^, ^12^, ^16^, ^17^, ^18^, ^20^

The integration of dual BCTs inside the eFLASH Mobetron allows redundant rapid dose monitoring and may allow energy monitoring based on the relative changes in the output between the upper and lower BCT due to differential attenuation of the electron beam with internal components (e.g., scattering foil and internal ion chambers) situated in between the upper and lower BCTs in the eFLASH Mobetron head. The lower‐to‐upper BCT ratio were found to provide detailed information regarding the energy of the eFLASH beam. This relationship can be used to evaluate the potential energy fluctuations on a pulse‐by‐pulse basis for both the 6‐MeV and 9‐MeV eFLASH beams. Even though individual variations in the BCT ratio were observed for each individual pulse in a 200‐pulse delivery, the pulse distribution tended to center on some nominal BCT ratio. Thus, the BCT ratio can be considered for the cumulative signal measured in the lower and upper BCT at a given pulse delivery, because the individual pulse values tended to be close together in their respective output with minimal variation. In AAPM TG‐72, recommendations for energy constancy in mobile electron accelerators were defined by an upper limit of 2‐mm in the shift in the R_50_.^33^ Here we investigated the utility of the BCT ratio as a novel method of monitoring energy in real time. The rationale was that the eFLASH beam output measured at the lower BCT would always be lower than that measured at the upper BCT because the internal ion chamber and secondary scattering foil would attenuate a portion of the primary beam. However, the amount of attenuation would depend strongly on the energy of the beam, as lower energy electron beams endure greater attenuation and scatter than higher energy electron beams. Overall, our results demonstrated that the BCT ratio can be used to monitor the energy of the eFLASH beam and is sensitive enough to detect distance shifts in R_50_ from 2‐mm to 3‐mm with sufficient statistical power. However, the statistical power for detecting shifts of 2‐mm was <90% for R_50_ values of 3–4 cm. This is most likely associated with limitations in how the energy of the beam was manipulated to acquire this data which was performed mainly through manipulating the gun grid voltage and the RF shorts position. With improved beam energy tuning and energy optimization, the data presented in Figure 7 would most likely demonstrate lower variation and stronger statistical power for R_50_ values of 3–4 cm than what we observed; *On par* with the high statistical power observed at both lower and higher R_50_ values, where the energy of the beam was closer to the nominal values and therefore better optimized. Daily energy monitoring using the BCT ratio for short‐ and long‐term stability, and further optimization in evaluating the energy of the beam with the BCT ratio, is currently being done to evaluate the full utility of BCT ratios as a means of energy monitoring in real time. It is important to note that individual BCT signals are shown to be dependent on the energy of the beam (Figure 7b) as evidenced by their decreasing sensitivity in signal per unit dose at higher beam energies. This is a consequence of the design of BCT integration into the head of the linac (energy dependent scatter conditions of the beam before going through the BCTs) and not of the BCTs themselves, which are energy independent. However, this inferred energy‐dependence of the BCTs in dose measurement and calibration emphasize the need for the user to perform routine energy checks and dose calibration of the beam to ensure accurate dosimetry in beam monitoring applications.

In this work, all data were acquired with the Mobetron situated in a room designed to shield for 18‐MV photons. A full radiation shielding survey, according to the recommendations in TG‐45^34^ and NCRP‐151,^35^ was obtained before the study was begun. This was deemed necessary because of the high doses per pulse and high number of pulses in a single delivery. The maximum number of pulses delivered was chosen based on the current limitation by the vendor when the system is run in Clinical mode. In a clinical context, 200 eFLASH pulses are unlikely to be used because of normal tissue dose constraints. However, a > 200‐pulse delivery is quite plausible in the context of quality assurance and commissioning purposes. We showed that accurate beam monitoring could be obtained independent of the number of pulses delivered.

One effect that must be taken under consideration in the use of BCTs for beam monitoring and control is the scatter and collimation conditions of the measurement setup. We found that differential contribution in electron scatter influences both the upper and lower BCT signal. More specifically, having a collimator with a smaller field size or having a scattering medium situated closer to the exit window of the beam influenced both the upper and lower BCT signal due to electrons backscatter, with the upper BCT signal being less sensitive to differences in the setup conditions. The lower BCT signal was observed to decrease (by as much as 12% relative to the open beam configuration), whereas the upper BCT signal was observed to vary slightly (by as much as 1.4% relative to the open beam configuration) when more material was obstructing the path of the FLASH electron beam, such as greater collimation restricting the beam‐path or when the exit window was moved closer to a scattering surface to facilitate electron scatter back into the head of the Mobetron. The reduction in signal in the lower BCT is most likely due to the greater backscatter with electrons traveling in the opposite direction of the beam delivered, thereby decreasing the net magnetic flux of the incident beam measured by the lower BCT. However, the reason for the increase in the signal from the upper BCT is unknown as the output of the eFLASH beam in its current configuration is not influenced by what is measured by the lower or upper BCT, thus requiring further investigation in future studies. The phenomenon of differential backscatter between different measurement setups is not specific to BCTs but is also highly prevalent in standard clinical linear accelerators, and the effects of electron backscatter on the measured BCT signal resemble the effects of monitor backscattering as reported in TG‐74^36^ and emphasize the need for electron output factors to correct for different scattering conditions also in this machine configuration.^17^ However, it should be noted that the tested configurations represents the extremes of what can be achieved in terms of electron backscatter. In any clinical situation, the maximum variation in setup would be change of field size and potentially change in air gap used. This would cause a maximum variation in measured signal of 1%–2% in the upper BCT, thus reducing the significance of this effect. Either way, proper use of output factors are recommended and the data also indicate a strong preference for using the upper BCT signal as the primary dose monitoring system because of its greater insensitivity to the setup conditions compared with the lower BCT.

## 5 CONCLUSIONS

This is the first detailed investigation of a dual‐BCT system design for monitoring and recording the individual pulse structure of an eFLASH beam and how this system is influenced by dose, dose rate, PW, PRF, scatter conditions, and DPP. It is important to emphasize that BCTs are a beam monitoring device that can measure the output of the eFLASH beam, and that their utility is in their use for relative dosimetry calibrated to specific dosimetric setups. We found that the BCTs’ responses were linear with respect to dose and DPP and no dependence on dose‐rate or PRF was found. The BCTs provided detailed information regarding the individual and cumulative pulse structure in a single delivery, which, when correlated with dose, can provide real‐time dosimetric information in a manner similar to the transmission ionization chambers in a conventional linear accelerator. Moreover, we found that the BCT ratio provided correlative information regarding the beam energy characteristics and can thus also be used for beam energy monitoring. Overall, the use of BCTs allow the beam to be monitored in real time without perturbation or saturation effects, which collectively allows highly detailed determinations of the physical beam parameters for each individual pulse delivered by UHDR FLASH irradiation. The fast signal readout and processing enables the BCTs to provide real‐time information on beam output and energy and is proposed as a system suitable for accurate beam monitoring and control of eFLASH beams.

## AUTHOR CONTRIBUTIONS

All authors were involved in the conceptualization, writing, editing, and figure visualization. Kevin Liu took the lead in data acquisition and initial data analyses. Emil Schüler and Sam Beddar were jointly responsible for project oversight, administration, and funding acquisition. All authors have read and agreed to the published version of the manuscript.

## CONFLICT OF INTEREST

None

## Supporting information

Supporting Information
