# Supplementary material for: Dual beam‐current transformer design for monitoring and reporting of electron ultra‐high dose rate (FLASH) beam parameters
Source: J Appl Clin Med Phys. 2023 Jan 4;24(2):e13891. doi: 10.1002/acm2.13891 (PMC9924113; doi:10.1002/acm2.13891)
Supplement: Supplementary file 1 — Supporting Information [file ACM2-24-e13891-s001.docx]

**SUPPLEMENTARY MATERIAL**

**Table S1.** Measured average signal per pulse from the upper beam current transformer (BCT), normalized to the single pulse delivery. Data is shown for different pulse widths (PWs) and at a constant pulse repetition frequency of 120 Hz.

|  | Pulse Widths | | |  |  |
| --- | --- | --- | --- | --- | --- |
| Number of Pulses | 1 µs | 1.2 µs | 2 µs | 3 µs | 3.6 µs |
| **1** | 1.00 | 1.00 | 1.00 | 1.00 | 1.00 |
| **2** | 0.99 | 1.00 | 0.98 | 1.00 | 0.98 |
| **5** | 0.98 | 1.00 | 0.97 | 0.99 | 0.97 |
| **10** | 0.98 | 0.99 | 0.97 | 0.98 | 0.97 |
| **20** | 0.97 | 0.99 | 0.96 | 0.97 | 0.96 |
| **50** | 0.96 | 0.96 | 0.94 | 0.95 | 0.94 |
| **100** | 0.96 | 0.97 | 0.95 | 0.96 | 0.95 |
| **200** | 0.97 | 0.98 | 0.96 | 0.98 | 0.96 |

**Table S2.** Measured average signal per pulse from the upper beam current transformer (BCT), normalized to the single pulse delivery. Data is shown for different pulse repetition frequencies at a constant pulse width of 1.2 µs.

|  |  | Pulse Repetition Frequency | | |
| --- | --- | --- | --- | --- |
| Number of Pulses | 30 Hz | 60 Hz | 90 Hz | 120 Hz |
| **1** | 1.00 | 1.00 | 1.00 | 1.00 |
| **2** | 1.00 | 1.00 | 1.00 | 1.00 |
| **5** | 0.99 | 1.00 | 0.99 | 1.00 |
| **10** | 0.99 | 1.00 | 0.99 | 0.99 |
| **20** | 0.99 | 0.99 | 0.99 | 0.99 |
| **50** | 0.98 | 0.99 | 0.98 | 0.96 |
| **100** | 0.98 | 0.99 | 0.99 | 0.97 |
| **200** | 0.98 | 0.98 | 0.99 | 0.98 |


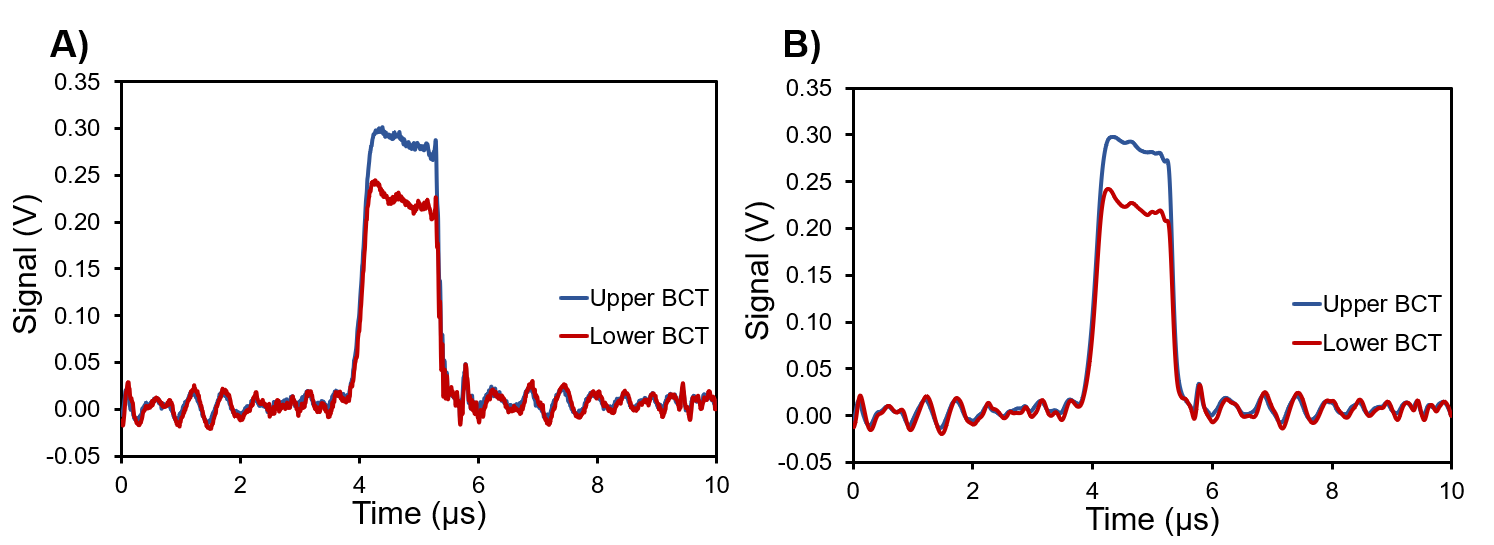


**Figure S1.** Visualization of the (A) measured signal (raw) and (B) the measured signal with a Bessel filter applied for a single pulse from a 9-MeV eFLASH beam at a pulse width of 1.2 µs for the upper and lower beam current transformer (BCT).


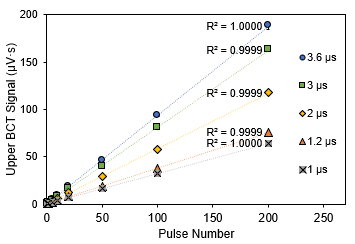


**Figure S2.** The measured signal from the upper beam current transformer (BCT) for pulse widths of 1 µs, 1.2 µs, 2, 3 µs, and 3.6 µs and a pulse repetition frequency of 120 Hz at delivered pulses of 1, 2, 5, 10, 20, 50, 100, and 200 pulses. The upper BCT signal was averaged over three readings at each delivered pulse number, with error bars representing one standard deviation from three measurements. Error bars are hidden behind their measurement point because of their relatively small value. The R^2^ values are presented for the trendlines produced at each pulse width.


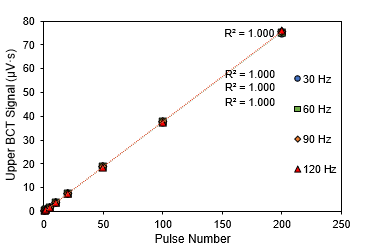


**Figure S3.** The measured signal from the upper beam current transformer (BCT) signal for a pulse width of 1.2 µs and for pulse repetition frequencies of 30 Hz, 60 Hz, 90Hz, and 120 Hz at delivered pulses of 1, 2, 5, 10, 20, 50, 100, and 200 pulses. The upper BCT signal was averaged over three readings at each delivered pulse number, with error bars representing one standard deviation from three measurements. Error bars are hidden behind their measurement point because of their relatively small value. The R^2^ values are shown for the trendlines produced at each pulse repetition frequency.
